# Metabolism and senescence in the immune microenvironment of osteosarcoma: focus on new therapeutic strategies

**DOI:** 10.3389/fendo.2023.1217669

**Published:** 2023-07-11

**Authors:** Hui Ying, Zhi-Qiang Li, Meng-Pan Li, Wen-Cai Liu

**Affiliations:** ^1^ Department of Emergency Trauma Surgery, Ganzhou People’s Hospital, Ganzhou, China; ^2^ Department of Spine Surgery, Ganzhou People’s Hospital, Ganzhou, China; ^3^ Department of Orthopaedic Surgery, The First Affiliated Hospital of Nanchang University, Nanchang, China

**Keywords:** osteosarcoma, metabolism, senescence, immune microenvironment, immunotherapy

## Abstract

Osteosarcoma is a highly aggressive and metastatic malignant tumor. It has the highest incidence of all malignant bone tumors and is one of the most common solid tumors in children and adolescents. Osteosarcoma tissues are often richly infiltrated with inflammatory cells, including tumor-associated macrophages, lymphocytes, and dendritic cells, forming a complex immune microenvironment. The expression of immune checkpoint molecules is also high in osteosarcoma tissues, which may be involved in the mechanism of anti-tumor immune escape. Metabolism and senescence are closely related to the immune microenvironment, and disturbances in metabolism and senescence may have important effects on the immune microenvironment, thereby affecting immune cell function and immune responses. Metabolic modulation and anti-senescence therapy are gaining the attention of researchers as emerging immunotherapeutic strategies for tumors. Through an in-depth study of the interconnection of metabolism and anti- senescence in the tumor immune microenvironment and its regulatory mechanism on immune cell function and immune response, more precise therapeutic strategies can be developed. Combined with the screening and application of biomarkers, personalized treatment can be achieved to improve therapeutic efficacy and provide a scientific basis for clinical decision-making. Metabolic modulation and anti- senescence therapy can also be combined with other immunotherapy approaches, such as immune checkpoint inhibitors and tumor vaccines, to form a multi-level and multi-dimensional immunotherapy strategy, thus further enhancing the effect of immunotherapy. Multidisciplinary cooperation and integrated treatment can optimize the treatment plan and maximize the survival rate and quality of life of patients. Future research and clinical practice will further advance this field, promising more effective treatment options for patients with osteosarcoma. In this review, we reviewed metabolic and senescence characteristics in the immune microenvironment of osteosarcoma and related immunotherapies, and provide a reference for development of more personalized and effective therapeutic strategies.

## 1 Introduction

Osteosarcoma is a highly aggressive bone tumor that originates as a malignant tumor in bone tissue, usually in adolescents and young adults, and can be extremely disruptive to the physical and mental health of patients (1–3). Osteosarcoma usually originates in the epiphysis of long bones, such as the femur, tibia and humerus, but can also occur in other skeletal sites. It is one of the most common malignant tumors in the skeletal system, accounting for approximately 20% of all bone tumors, and is prone to recurrence and early lung metastases (1, 4, 5). The tumor cells of osteosarcoma are highly proliferative and infiltrative, often rapidly invading surrounding bone tissue and adjacent structures, leading to bone destruction and functional impairment. The treatment of osteosarcoma is complicated by its highly aggressive nature and early onset of pulmonary metastasis (6, 7). Despite the current comprehensive treatment strategies including surgical resection, radiotherapy and chemotherapy, the prognosis of osteosarcoma is still not optimistic (8–10).

**Figure 1:**
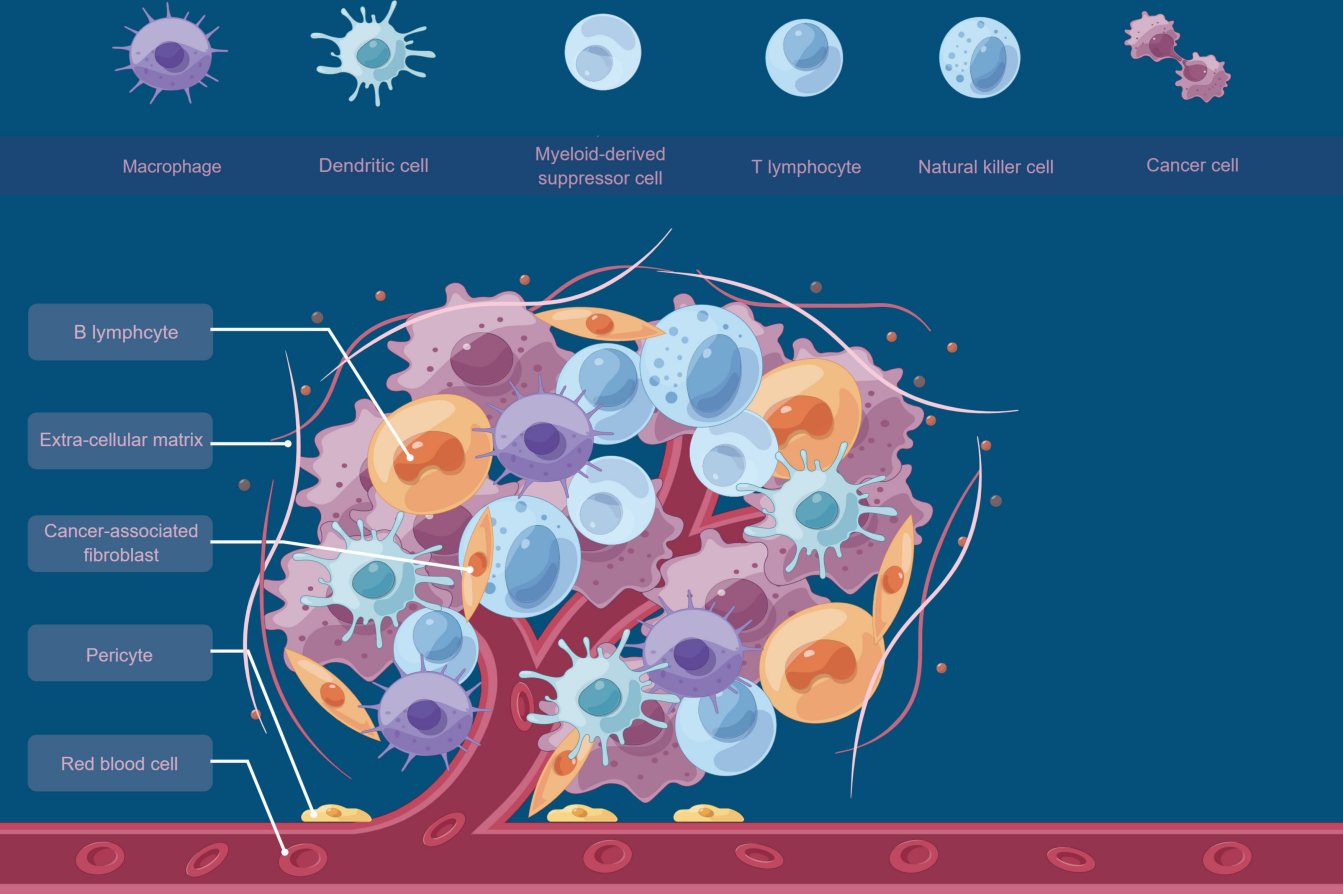
A brief overview of the tumor microenvironment.

Recent studies have shown that the immune microenvironment plays a key role in the development and progression of osteosarcoma (11–13), and **Figure 1** briefly shows an overview of the tumor microenvironment. Osteosarcoma tissues are often richly infiltrated with inflammatory cells, including tumor-associated macrophages, lymphocytes, and dendritic cells, forming a complex immune microenvironment (3, 14). The expression of immune checkpoint molecules such as PD-L1 and CTLA-4 is also high in osteosarcoma tissues, which may be involved in the mechanism of anti-tumor immune escape (15–17). Osteosarcoma cells evade recognition and attack by the immune system in several ways. On the one hand, osteosarcoma cells can reduce the expression of antigens and decrease the chance of attack by immune cells. On the other hand, osteosarcoma cells can also produce immunosuppressive factors, such as immune checkpoint inhibitors and immunosuppressive cells, thereby inhibiting the activity of immune cells and reducing the attack on tumor cells (18, 19). In addition, the inflammatory response can lead to infiltration and activation of immune cells, releasing a variety of cytokines and chemokines that stimulate the growth, invasion and metastasis of tumor cells (20–22). It can also lead to increased expression of antigens by tumor cells, thus increasing recognition and attack by the immune system. Besides immune cell infiltration also plays an important role in osteosarcoma. It has been found that the type, number and activity of immune cells in the tumor microenvironment have an important impact on the growth and metastasis of osteosarcoma (23, 24). Immune cell infiltration can contribute to an immune attack of tumor cells, but at the same time, immune cells may also be suppressed by immunosuppressive factors produced by tumor cells, thus limiting the effect of the immune system on the tumor.

Metabolism and senescence are closely related to the immune microenvironment, and disturbances in metabolism and senescence may have important effects on the immune microenvironment, thereby affecting immune cell function and immune response (25–27). Metabolic disorders affect the biological activity and function of immune cells by altering their energy metabolic pathways, such as glycolysis and mitochondrial respiration. In addition, the accumulation of metabolites may affect the signaling pathways such as endoplasmic reticulum stress and oxidative stress in immune cells, thus affecting immune regulation and anti-inflammatory responses of immune cells (22, 28–30). For senescence, age-related biological changes may lead to alterations in the quantity and quality of immune cells, thus affecting the immune response and the balance of the immune microenvironment. In addition, senescence may lead to alterations in surface markers, antigen presentation and signaling of immune cells, thus affecting the function of immune cells and the effectiveness of the immune response (25, 26, 31).

In this review, we reviewed the metabolic and senescence characteristics in the immune microenvironment of osteosarcoma and related immunotherapies, with the aim of providing a reference for studying the interconnection of metabolism and senescence in the immune microenvironment of osteosarcoma, the mechanisms regulating immune cell function and immune response, and the development of more precise therapeutic strategies.

## 2 Metabolic characteristics of the immune microenvironment in osteosarcoma

### 2.1 Mechanism of interaction between metabolism and immune regulation

There is a complex mechanism of interaction between tumor metabolism and immune regulation. The metabolic pathways and metabolites of tumor cells can directly or indirectly affect the function of immune cells and immune regulation, thus having an impact on the immunotherapy of tumors (28, 32–34).

The metabolic pathways of tumor cells can affect the biological activity of immune cells (35). Tumor cells often exhibit high levels of glycolysis and lactate production, leading to low pH and hypoxic conditions in the tumor microenvironment (36). These metabolic features affect immune cell function, such as altering immune cell growth, proliferation, antigen recognition and antigen presentation capabilities. In addition, the metabolic activity of tumor cells leads to the accumulation of some metabolites, such as lactate, adenosine and pyruvate, which have an immunosuppressive effect on immune cells and thus attenuate the immune response (37).

Metabolites of tumor cells can modulate the immune response by altering the signaling pathways of immune cells (35, 38). For example, metabolites such as lactate and adenylate can inhibit immune cell function by binding to the corresponding receptors on immune cells (39, 40). Adenosine and adenosine receptors may play an important immunosuppressive role in the tumor microenvironment, affecting immune cell proliferation, differentiation, antigen recognition and antigen presentation. In addition, some metabolites such as pyruvate and ketone bodies can also modulate the immune response by affecting the metabolic pathways and signaling pathways of immune cells (41).

The metabolic pathways and metabolites of tumor cells can also indirectly affect immune regulation by altering the composition and function of immune cells in the tumor microenvironment (11, 35, 42). For example, the metabolic profile of tumor cells may lead to alterations in the number and activity of immune cells in the tumor microenvironment, such that the functions of T cells, natural killer cells (NK cells), and dendritic cells can be suppressed, thus weakening the immune system’s ability to attack the tumor (43, 44). In addition, metabolites of tumor cells can also have an impact on immune tolerance of immune cells, leading to a decrease in the ability of immune cells to recognize and attack tumor cells.

Osteosarcoma tumor cells exhibit metabolic specificities such as increased glycolysis, altered mitochondrial function, and increased lactate production. These metabolic alterations produce an acidic, hypoxic, and nutrient-deprived microenvironment (45). The acidic microenvironment can inhibit the function of immune cells such as T cells and natural killer cells by altering the pH-sensitive signaling pathways that are necessary for their activation and proliferation (46). For example, the acidic microenvironment can inhibit the activity of enzymes involved in T cell receptor signaling, leading to impaired T cell activation and cytokine production (47). Additionally, the acidic microenvironment can promote the survival and proliferation of immunosuppressive cells such as regulatory T cells and myeloid-derived suppressor cells, which can further inhibit the function of infiltrating immune cells (48). Hypoxia can promote the expression of immune checkpoint molecules such as PD-1 and CTLA-4, which can inhibit the function of infiltrating immune cells and promote immune escape of osteosarcoma cells (49). In a nutrient-poor microenvironment, immune cells may become metabolically stressed and unable to carry out their normal functions. This can result in decreased cytokine production, impaired cytotoxicity, and reduced activation and proliferation of immune cells (42). Additionally, nutrient deprivation can lead to the activation of stress response pathways, such as the unfolded protein response (UPR) and the integrated stress response (ISR), which can further impair immune cell function and promote the survival of osteosarcoma cells (50).

### 2.2 Metabolic regulatory mechanisms of the immune microenvironment in osteosarcoma

#### 2.2.1 Glucose metabolism regulation

Osteosarcoma cells are highly glucose-dependent and their metabolic pathways include mainly glycolysis and gluconeogenesis (51). In the immune microenvironment, osteosarcoma cells escape from immune cells by regulating glycolytic pathways. For example, osteosarcoma cells can promote glycolysis to produce lactate through the high expression of key glycolytic enzymes such as phosphofructokinase (PFK) and lactate dehydrogenase A (LDHA), thereby allowing immune cells to form an acidic microenvironment around the tumor and inhibit the killing function of immune cells (52–55). In addition, osteosarcoma cells can also meet their own energy and biosynthetic needs and reduce the energy supply to immune cells by increasing glucose production from the gluconeogenic pathway (51).

#### 2.2.2 Ammonia metabolism regulation

Osteosarcoma cells typically exhibit a high degree of ammonia metabolism, with metabolic pathways including ammonia degradation and glutamate metabolism (56). In the immune microenvironment, osteosarcoma cells influence the function of immune cells by regulating ammonia metabolic pathways (57, 58). For example, osteosarcoma cells can inhibit immune cell activity by increasing the expression of glutamate acidifying enzyme (GLUD1), which drives the conversion of ammonia to glutamate. In addition, ammonia can inhibit the metabolic activity of immune cells by activating ketoacid dehydrogenase (BCAT1) in immune cells, thereby reducing the attack of immune cells on tumor cells (59, 60).

#### 2.2.3 Lipid metabolism regulation

Regulation of lipid metabolism by osteosarcoma cells in the immune microenvironment affects the function of immune cells mainly by regulating processes such as lipid acid synthesis, storage and oxidation (61, 62). For example, osteosarcoma cells can increase lipid acid synthesis and storage by increasing lipid acid synthases such as fatty acid synthase (FASN) and lipoyl-CoA synthase (ACSL), which provide energy and raw materials required for tumor cell growth and biosynthesis (63). In addition, osteosarcoma cells can also reduce the oxidative metabolic activity of immune cells by inhibiting lipid acid oxidase enzymes such as hydroxy acyl-coenzyme A dehydrogenase (HADHA) and hydroxy acyl-coenzyme A lyase (HADHB) in immune cells, thereby weakening the ability of immune cells to attack tumor cells (63, 64).

#### 2.2.4 Oxidative stress regulation

Osteosarcoma cells produce large amounts of reactive oxygen species (ROS) when they are in a state of high oxidative stress. In the immune microenvironment, osteosarcoma cells influence the function of immune cells by regulating the production and clearance of ROS (65–67). For example, osteosarcoma cells can increase ROS production by increasing the expression of ROS-producing enzymes such as members of the NADPH oxidase (NOX) family. These ROS can lead to impaired immune cell function by directly oxidizing key proteins within the immune cells. In addition, ROS can also inhibit the immune effects of immune cells by regulating their signaling pathways and transcription factor activity (68–71).

#### 2.2.5 Immune cell metabolism regulation

**Figure 2:**
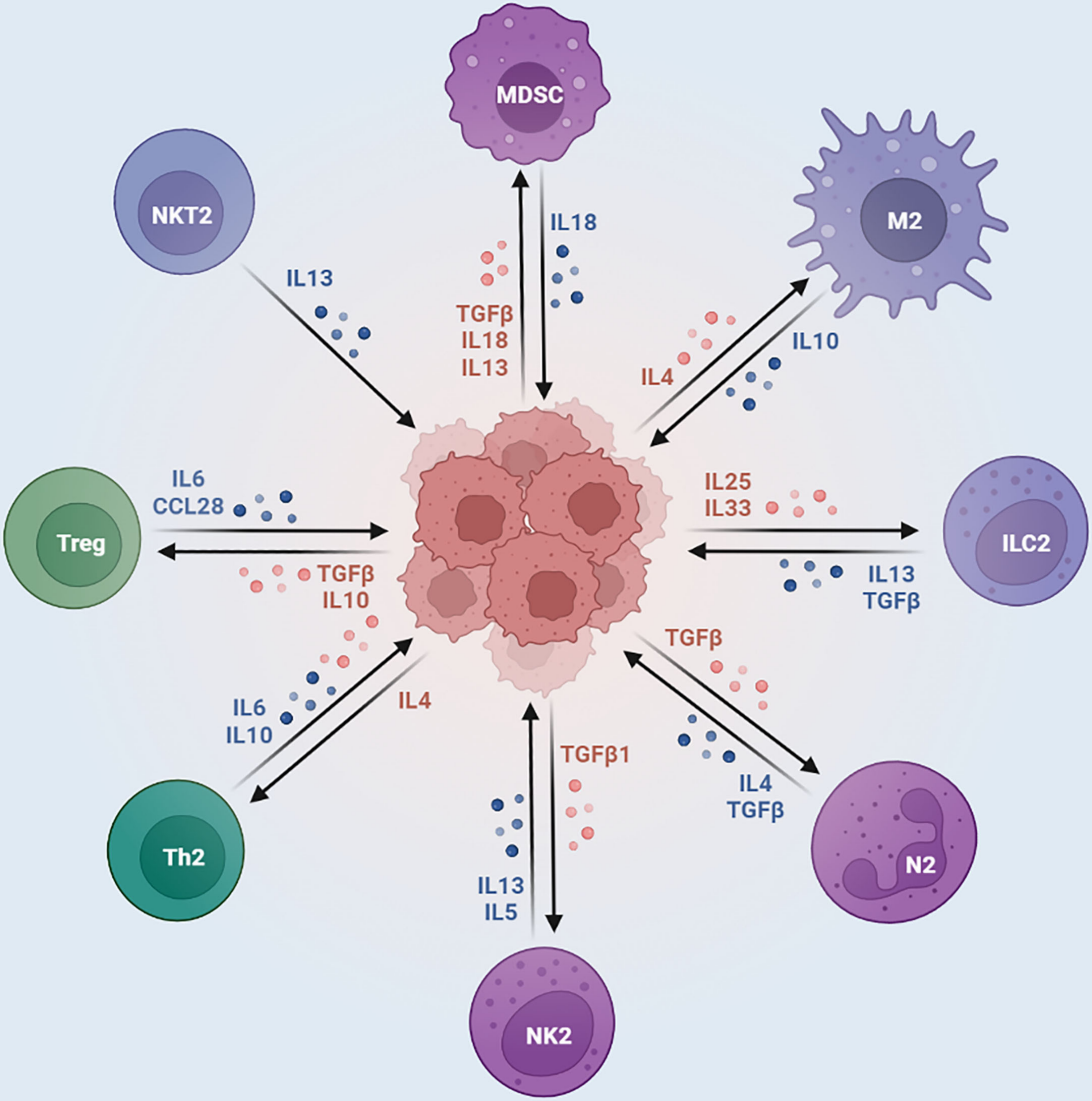
Immunosuppressive Cells in the Tumor Microenvironment.

The ability of immune cells in the immune microenvironment to attack tumor cells is also influenced by metabolic modulation (32, 72). For example, immune cells in the tumor microenvironment such as cytotoxic T lymphocytes (CTLs) and natural killer cells (NK cells) play a key role in antitumor immunity (73). The metabolic state of these immune cells in the immune microenvironment is regulated by osteosarcoma cells. For example, osteosarcoma cells can reduce the metabolic activity of immune cells by releasing metabolic products such as lactate and adenylate. Lactate can reduce the energy supply of immune cells by inhibiting oxidative phosphorylation of mitochondria within immune cells, thereby reducing their antitumor activity (74). Adenosine can suppress tumor immune response by activating adenosine receptors on immune cells, leading to the suppression of immune cells and an increase in immune antigen-specific T cells (Tregs) (75, 76). **Figure 2** describes the bidirectional interactions between tumor cells and the immunosuppressive component of the tumor microenvironment.

## 3 Senescence characteristics of the immune microenvironment in osteosarcoma

### 3.1 Concepts and mechanisms of immune senescence

Immunosenescence is the phenomenon of gradual decline in the function of the human immune system with age, which is mainly manifested by a decrease in the body’s immune response to endogenous and exogenous antigens, a lack of responsiveness to new antigens, and a blunted response to the protective effects of vaccines and established immune memory, resulting in a decrease in the individual’s ability to defend against infectious diseases, anti-tumor capacity, and the ability to clear senescent cells with age (77–81). It is a physiological phenomenon, but may also accelerate the onset and progression of disease. As an epidemiological study of osteosarcoma shows that the second peak in osteosarcoma is over 65 years of age (82). The age of the patient is correlated with the survival, with the poorest survival among older patients (82, 83). Therefore, the exploration of the mechanism of age-related immune senescence may provide new ideas for the treatment of this population.

Immunosenescence is associated with a decrease in the number and function of immune cells (84–86). With aging, there is a gradual decrease in the number and activity of immune cells in the body, including T cells, B cells, natural killer cells, and other immune cells. This may lead to a weakened immune response of the body to pathogens and tumor cells, thus increasing the risk of infection and tumor development.

Alterations in immune cell function also play a role in immunosenescence (84, 85, 87). With aging, immune cell functions are affected, including antigen presentation, antibody production, and cytotoxic activity. For example, T cells in the elderly may exhibit reduced proliferation and activity and a diminished response to novel antigenic stimuli, resulting in diminished immune memory and a decreased antigen-specific immune response.

Immunosenescence is related to altered inflammatory status (88). With advancing age, the body may develop a chronic inflammatory state called “inflammatory aging”. This chronic inflammatory state leads to an excessive inflammatory response of immune cells in response to pathogens and tumor cells, thus affecting the function and regulation of immune cells.

Alterations in immune regulatory networks also have an impact on immunosenescence (89–91). The normal function of the immune system depends on complex immune regulatory networks, including the interactions between immune cells, the production and action of cytokines, and the signaling between immune cells and target cells. As we age, these immune regulatory networks may change, leading to abnormal immune responses and disruption of immune tolerance.

### 3.2 Effects of the senescence characteristics in osteosarcoma immune microenvironment

With aging, the immune microenvironment of osteosarcoma will exhibit some senescence characteristics, which will have an impact on the immune response and disease process of osteosarcoma (31, 77, 92). As the aging process of the body continues to progress, the number and function of immune cells in the body will decline, including T cells, B cells, natural killer cells, and other immune cells (84, 87, 93, 94). This will lead to a weakened immune response in patients with osteosarcoma, which will increase the escape and survival of tumor cells. The function of immune cells will also be affected, including antigen presentation, antibody production, and cytotoxic activity. T cells will show reduced proliferation and activity, and a diminished response to new antigenic stimuli, resulting in a decreased immune response. At the same time, the immune microenvironment in osteosarcoma can develop a chronic inflammatory state (88, 95, 96). This chronic inflammatory state leads to an excessive inflammatory response of immune cells in response to tumor cells, which affects the function and regulation of immune cells. In addition, chronic inflammation may also promote the survival and proliferation of tumor cells (97). The immune regulatory network in the immune microenvironment of osteosarcoma will also be altered, including the interaction between immune cells, the production and action of cytokines, and the signaling between immune cells and tumor cells (98). This will lead to abnormal immune response and disruption of immune tolerance, thus affecting the immune surveillance and anti-tumor immune response to tumor cells in patients with osteosarcoma

**Figure 3:**
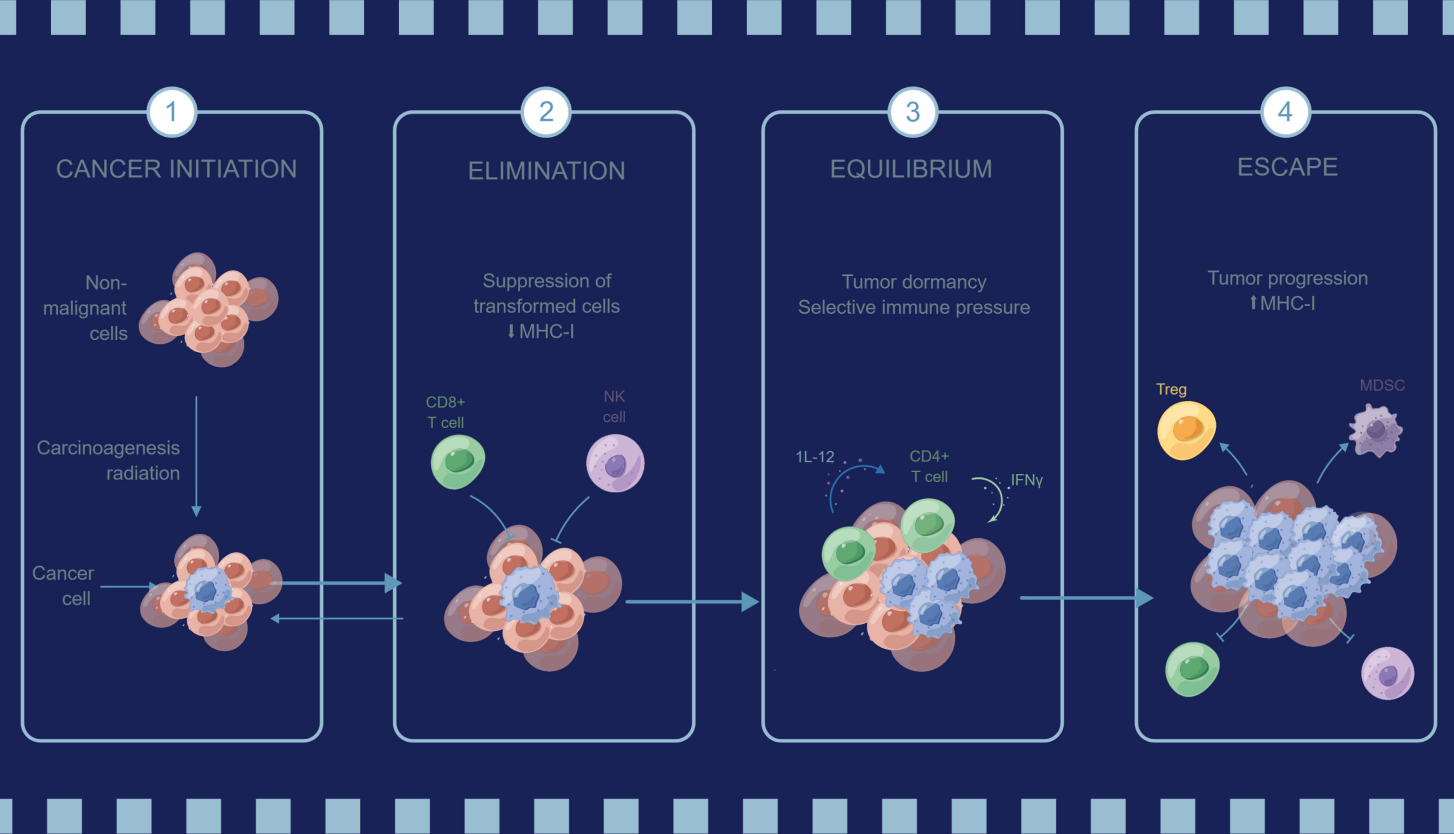
A brief overview of tumor immune evasion mechanism.

With aging of the immune microenvironment, tumor cells can escape from immune surveillance by various mechanisms, such as reduced antigen presentation and antigen expression, altered evasive expression of antigens, and reduced recognition and killing of tumor antigen-specific T cells, thereby shielding tumor cells from immune attack (85, 86, 93). **Figure 3** briefly describes the process mechanism of cancer initiation, elimination, equilibrium and escape under the influence of immune cells in the immune microenvironment. Senescence of the immune microenvironment may also lead to disruption of immune tolerance mechanisms, enabling tumor cells to escape clearance in the immune system, thus promoting tumor cell survival and proliferation (99). Senescence of the immune microenvironment may also lead to an increase in immunosuppressive factors, such as an increase in anti-inflammatory cytokines and an increase in immunosuppressive cells, which may inhibit the activity and function of immune cells and affect the antitumor immune response (100). Aging may lead to a decrease in the memory function of immune cells, thus reducing the specific attack on osteosarcoma cells (101). Decreased immune memory may lead to a reduced ability of immune cells to re-attack osteosarcoma cells, thus affecting the effectiveness of immunotherapy (102).

The immune microenvironment of osteosarcoma exhibits features such as diminished immune cell numbers and functions, altered immune regulatory networks, and altered inflammatory status during aging, which affect the immune response and disease process in osteosarcoma (103). Several studies have shown that intervening in the senescence features of the immune microenvironment in osteosarcoma (104), may help to enhance the effectiveness of immune cell attack on osteosarcoma cells and thus improve the efficacy of immunotherapy (105–107). For example, some studies have shown that by inhibiting immunosuppressive pathways such as PD-1/PD-L1 and CTLA-4, the ability of immune cells to attack osteosarcoma cells can be restored, thereby improving the efficacy of immunotherapy (15, 16, 108–110).

## 4 Strategies against immunosenescence or metabolic regulation

### 4.1 Compounds and drugs against immunosenescence

Several compounds and drugs are used to counteract immunosenescence, the age-related decline in immune function. Some of the most common ones include: 1) Thymosin alpha 1 (Tα1): Tα1 is a peptide that has been shown to enhance T-cell function and modulate the immune response (111). It can improve the production and maturation of T cells, which decline with age, thus counteracting immunosenescence (112). 2) Interleukin-7 (IL-7): IL-7 is a cytokine that plays a crucial role in the development and maintenance of T cells (113). Recombinant IL-7 therapy has been shown to increase T cell numbers and improve immune function in aged individuals (114). 3) PD-1/PD-L1 inhibitors: These drugs, such as pembrolizumab and nivolumab, target the immune checkpoint proteins PD-1 and PD-L1 (115). These proteins are often upregulated in aged immune cells and contribute to immune senescence. By blocking the interaction between PD-1 and PD-L1, these inhibitors can enhance the immune system’s ability to recognize and attack pathogens and cancer cells. 4) CTLA-4 inhibitors: Ipilimumab is a monoclonal antibody that targets the immune checkpoint protein CTLA-4 (116). CTLA-4 is expressed on the surface of T cells and acts as a negative regulator of T cell activation (117). By blocking CTLA-4, ipilimumab can enhance T cell activation and promote a more robust immune response (118). 5) Senolytics: These compounds selectively target and eliminate senescent cells, which accumulate with age and contribute to immunosenescence (119). Examples of senolytics include dasatinib, quercetin, and navitoclax. By removing senescent cells, senolytics can reduce inflammation and improve immune function (120). 6) Rapamycin (sirolimus): Rapamycin is an immunosuppressive drug that specifically targets the mTOR pathway (121). By inhibiting the mTOR pathway, rapamycin can suppress age-related changes in immune cells and improve immune function, although its use as an anti-immunosenescence agent should be approached with caution due to its immunosuppressive properties (122). These drugs and compounds have different mechanisms of action, but they all aim to improve immune function and counteract immunosenescence.

### 4.2 Compounds and drugs of metabolic regulation

Several compounds and drugs can be used for metabolic regulation of the immune microenvironment of tumor cells. Some of the most common ones include: 1) Metformin: Metformin is a widely used drug for treating type 2 diabetes. It has been shown to have anti-cancer properties by modulating the tumor microenvironment (123). Metformin activates AMP-activated protein kinase (AMPK), which regulates cellular metabolism and inhibits the mammalian target of rapamycin (mTOR) pathway (124). This can lead to reduced nutrient availability for tumor cells and improved immune cell function within the tumor microenvironment (125). 2) 2-Deoxy-D-glucose (2-DG): 2-DG is a glucose analog that inhibits glycolysis, a key metabolic pathway used by cancer cells for energy production (126). By inhibiting glycolysis, 2-DG can alter the metabolic landscape of the tumor microenvironment, making it less favorable for tumor growth and more supportive of immune cell infiltration and function (127). 3) Dichloroacetate (DCA): DCA is a small molecule that targets pyruvate dehydrogenase kinase (PDK), an enzyme involved in the regulation of glucose metabolism. By inhibiting PDK, DCA can shift cancer cell metabolism from glycolysis to mitochondrial oxidative phosphorylation, reducing lactate production and acidification of the tumor microenvironment (128). This can improve immune cell function and enhance anti-tumor immunity. 4) Indoleamine 2,3-dioxygenase (IDO) inhibitors: IDO is an enzyme that degrades the essential amino acid tryptophan, leading to immunosuppression within the tumor microenvironment (129). IDO inhibitors, such as epacadostat and indoximod, can restore tryptophan levels and improve immune cell function, promoting anti-tumor immunity (130). 5) Adenosine receptor antagonists: Adenosine is an immunosuppressive molecule that accumulates in the tumor microenvironment (131). Adenosine receptor antagonists, such as CPI-444 and AZD4635, can block the interaction between adenosine and its receptors on immune cells, enhancing immune cell activation and anti-tumor responses (132). 6) CD73 inhibitors: CD73 is an enzyme that generates adenosine in the tumor microenvironment, contributing to immunosuppression (133). CD73 inhibitors, such as oleclumab and NZV930, can block adenosine production and improve immune cell function, promoting anti-tumor immunity (134). These compounds and drugs target different aspects of tumor cell metabolism and the immune microenvironment, aiming to improve immune cell function and promote anti-tumor responses. It is important to note that some of these compounds are still under investigation, and their long-term safety and efficacy have not been fully established.

## 5 Therapeutic strategies for the immune microenvironment of osteosarcoma

### 5.1 Current status and challenges of immunotherapy in osteosarcoma

As a highly aggressive tumor, traditional treatments for osteosarcoma include surgery, radiotherapy and chemotherapy, but their efficacy remains limited in advanced or recurrent osteosarcoma (135–137). Therefore, in recent years, immunotherapy has attracted much attention as a new treatment strategy (1, 105). Immunotherapy uses the body’s immune system to attack tumor cells by enhancing the ability of immune cells to recognize and attack tumors. In osteosarcoma, immunotherapy mainly includes immune checkpoint inhibitors, tumor vaccines, cellular immunotherapy and gene editing immunotherapy.

Currently, immune checkpoint inhibitors have made some progress in the clinical application of osteosarcoma as the most used immunotherapeutic agents (1, 72, 107, 138, 139). Immune checkpoint inhibitors, such as PD-1, B7-H3 and CTLA-4 inhibitors, enhance the ability to attack tumors by relieving the suppression of immune cells and activating the body’s immune response (104, 108, 110, 140, 141). Several clinical trials have shown that immune checkpoint inhibitors have achieved some clinical efficacy in a subset of patients with osteosarcoma (108, 142–147). However, overall, the sensitivity of osteosarcoma to immune checkpoint inhibitors is not high, and further validation of the therapeutic effect is needed (140, 147, 148).

Tumor vaccines have also shown potential as an individualized immunotherapeutic strategy in osteosarcoma (149, 150). Tumor vaccines can stimulate a tumor-specific immune response by directing the body’s immune system to recognize and attack tumor cells. Tumor vaccines are mainly classified as autologous cancer and immune cell-based vaccines and non-cell-based vaccines (149, 151, 152). Among these types, immune cell-based vaccines make full use of innate immune cells to activate effector T lymphocytes (153, 154). However, the feasibility of regulating migration and activation is a major issue, as these processes are regulated by immunosuppressive substances in the tumor microenvironment as well as by the quantity and quality of the patient’s immune effector cells (149).

**Figure 4:**
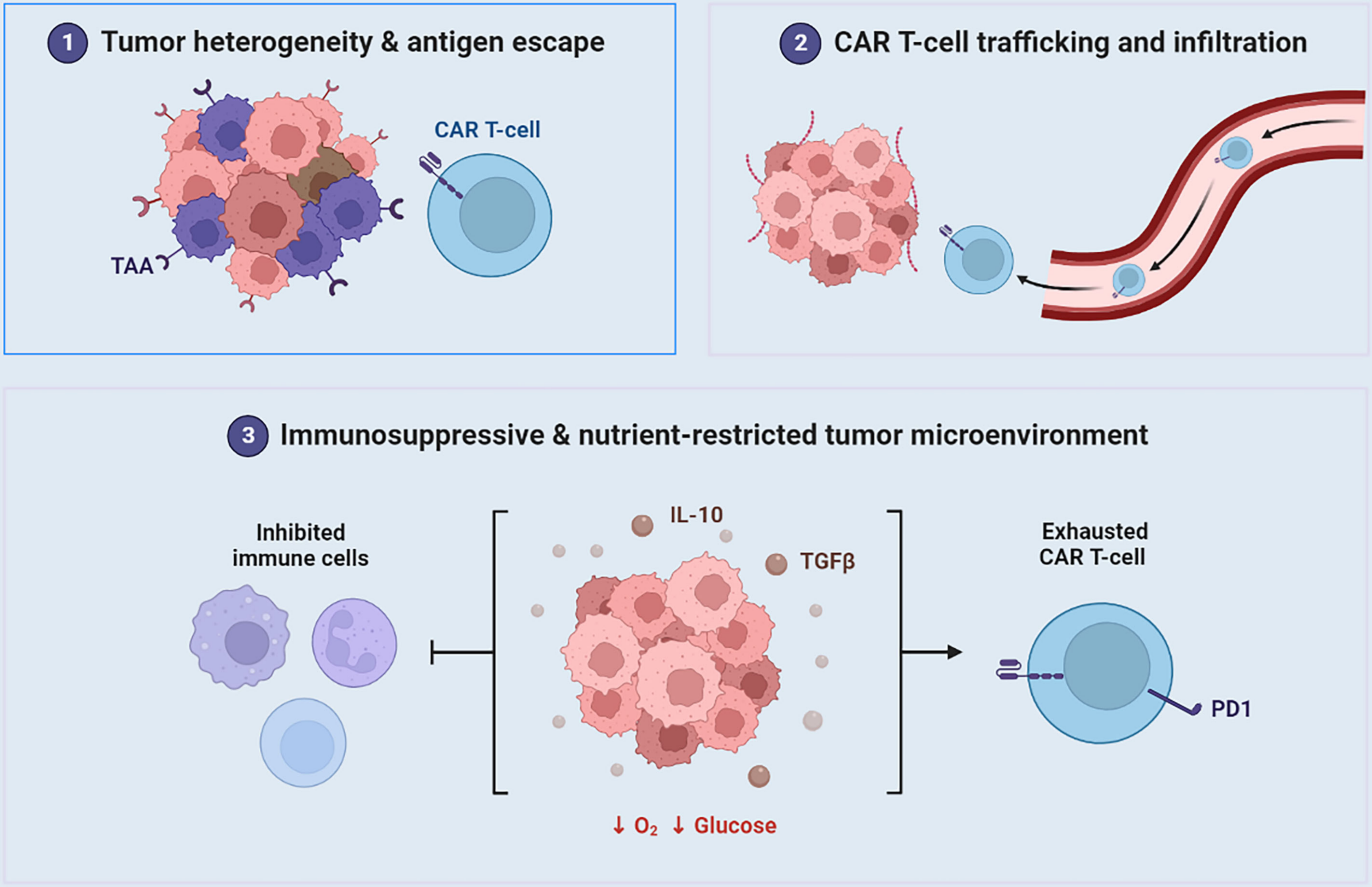
Challenges for CAR T-cell Immunotherapy in Solid Tumors (1). heterogeneous expression of tumor-associated antigens (TAA), leading to the growth of antigen-negative tumor variants (2); inefficient trafficking of CAR T cells at tumor sites (3); a poorly metabolized tumor microenvironment, including immunosuppressive molecules and cells are present, which can lead to CAR T-cell exhaustion.

Cellular immunotherapy, such as CAR-T cell therapy, has achieved remarkable efficacy in some tumors, especially hematologic tumors, but its application in solid tumor such as osteosarcoma remains challenging (**Figure 4**) (155–161). The complex immune microenvironment of osteosarcoma and the high heterogeneity of tumor cells have limited the application of cellular immunotherapy in osteosarcoma (158, 162). In addition, there are certain resistance mechanisms of osteosarcoma cells to CAR-T cell attack, including T cell suppression, cytokine tolerance, and immune escape from the tumor microenvironment (158, 163).

Gene editing immunotherapy is an emerging immunotherapeutic strategy that includes technologies such as CRISPR-Cas9 that can target specific genes in tumor cells for editing, thereby enhancing the ability of immune cells to attack tumors (161, 164–166). Although gene editing immunotherapy in osteosarcoma is still in its early stages of research, it has made some breakthroughs in treating other types of tumors, offering new hope for immunotherapy of osteosarcoma (166–168).

## 6 Conclusions and perspectives

Osteosarcoma is a highly aggressive osteosarcoma with an immune microenvironment that plays an important role in tumor growth and progression. At present, many compounds and drugs have been found to interfere with immune senescence and metabolic regulation of tumor cells. Metabolic modulation and anti-senescence therapy are gaining the attention of researchers as emerging immunotherapeutic strategies for tumors.

However, there are still some challenges in the application of metabolic modulation and anti-senescence therapy in osteosarcoma. These include the lack of sufficient clinical trial data and long-term follow-up data, and the safety and efficacy of the treatment are yet to be validated; the selection and optimization of treatment strategies still need further research; the complexity and diversity of the tumor immune microenvironment lead to individual differences and requires personalized treatment approaches; and the cost and feasibility of treatment also need to be considered.

Future research could explore the following directions to advance the prospects of metabolic modulation and anti-senescence therapies in the immune microenvironment of osteosarcoma:1) Clinical trial design and implementation: Conduct more clinical trials to validate the safety, efficacy and long-term efficacy of metabolic modulation and anti-aging therapies in osteosarcoma patients. Obtain more reliable clinical data through multi-center, large sample clinical trials to further clarify the optimization and application of treatment strategies. 2) In-depth study on the metabolic regulation mechanism of immune cells: In-depth study on the metabolic regulation mechanism of immune cells in the immune microenvironment of osteosarcoma, revealing the role of different metabolic pathways in immune cell function and immune response, and providing the theoretical basis for the development of new metabolic regulation strategies. 3) Personalized treatment strategies: Considering the complexity of the tumor immune microenvironment and individual differences, develop personalized treatment strategies, and select appropriate metabolic regulation and anti-aging therapies according to patients’ pathological characteristics, immune status and metabolic status to improve treatment effects.4) Multidisciplinary cooperation and integrated treatment: The application of metabolic modulation and anti-aging therapy in the immune microenvironment of osteosarcoma requires multidisciplinary cooperation, including experts in oncology, immunology and metabolism, to integrate different therapeutic approaches and form an integrated treatment strategy to maximize the therapeutic effect. 5) Biomarker screening and application: Research and application of biomarkers for screening patients suitable for metabolic modulation and anti-senescence therapy, monitoring treatment effects and predicting efficacy, thus guiding clinical practice.

Overall, metabolic modulation and anti- senescence therapies show potential application in the immune microenvironment of osteosarcoma. Future research and clinical practice will further advance this field and hopefully, provide more effective treatment options for osteosarcoma patients.

## Author contributions

W-CL and HY conceived of and designed the study. M-PL and W-CL generated the figures. Z-QL, M-PL, HY, and W-CL wrote the manuscript and Z-QL, W-CL and HY critically reviewed the manuscript. All authors contributed to the article and approved the submitted version.
